# Double-stranded RNA adenosine deaminase ADAR1 enhances both T cell susceptibility to human T-cell leukemia virus type 1 and 2 and viral replication

**DOI:** 10.1186/1742-4690-11-S1-O49

**Published:** 2014-01-07

**Authors:** Anne Cachat, Sébastien A Chevalier, Sandrine Alais, Adrien Boniface, Nga Ling Ko, Antoine Gessain, Hélène Dutartre, Renaud Mahieux

**Affiliations:** 1 Oncogenèse Rétrovirale, Equipe labellisée Ligue nationale contre le cancer, CIRI, INSERM U1111-CNRS UMR5308, Université Lyon 1, Ecole Normale Supérieure, LabEx ECOFECT - Eco-evolutionary dynamics of infectious diseases, Lyon, Cedex 07, France; 2 Unité d'Epidémiologie et Physiopathologie des Virus Oncogènes, CNRS URA 3015, Institut Pasteur, Paris, Cedex 15, France

## 

Type I interferons represent the first line of defense against pathogens. This family of cytokines activates the expression of antiviral proteins, such as the protein kinase R (PKR), an inhibitor of viral mRNA translation, and the double-stranded RNA adenosine deaminase ADAR1. ADAR1 has the ability to convert adenosine (A) into guanosine (G), thereby introducing mutations in the viral genome during its replication. A to G editing was previously reported in cells expressing HTLV-2 or STLV-3 viruses but not investigating in HTLV-1 expressing cells (Ko et al. J. Gen Virol. 2013). Consequently we investigated whether ADAR1 expression was associated or not with an antiviral effect in the course of HTLV-1 and HTLV-2 infections. We first show that ADAR1 expression is increased in ATL patient peripheral blood mononuclear cells, in HTLV-1 and HTLV-2 transformed cell lines as well as in activated primary peripheral blood lymphocytes. Strikingly, in cells transfected with HTLV-1 and HTLV-2 molecular clones, ADAR1 over-expression enhances viral replication and viral egress through PKR functional inhibition, as demonstrated by western-blot analyses, luciferase assays, ELISA and infection experiments. We also demonstrate that this effect is independent of ADAR catalytic activity. In addition, ADAR1 expression enhances the susceptibility of a non-infected T cell line to HTLV-1 and HTLV-2 infection. Altogether, our results demonstrate that an interferon-induced protein exerts a proviral role in the context of HTLV infection by enhancing cells susceptibility to infection and increasing viral replication.

